# Differential hypothalamic regulation of FSH and LH secretion from the fish pituitary by GnRH and CCK

**DOI:** 10.1530/REP-25-0182

**Published:** 2025-10-28

**Authors:** Naama Mizrahi, Miriam Shulman, Tomer Aiznkot, Ishwar Atre, Hadar Mor, Lian Hollander-Cohen, Berta Levavi-Sivan

**Affiliations:** Department of Animal Sciences, The Robert H Smith Faculty of Agriculture, Food, and Environment, Hebrew University of Jerusalem, Rehovot, Israel

**Keywords:** GnRH receptor, CCK receptor, feed intake, ovarian development

## Abstract

**In brief:**

The hypothalamus–pituitary–gonad axis integrates environmental and internal signals to tune reproductive functions. We hypothesized that in fish, Gnrh predominantly stimulates luteinizing hormone (LH) secretion, while Cck selectively regulates follicle-stimulating hormone (FSH), thereby uncovering a novel role for Cck as a metabolic gatekeeper that links reproductive activity with nutritional status.

**Abstract:**

In fish, the hypothalamus–pituitary–gonad axis governs reproduction in response to environmental and internal signals. FSH regulates gonadal growth, while LH controls maturation. Gonadotropin-releasing hormone (Gnrh) plays a key role in this process, while the role of the satiety hormone cholecystokinin (Cck) is under investigation. We hypothesized that Gnrh and Cck differentially regulate tilapia LH and FSH, reflecting distinct reproductive and metabolic roles. Gnrh receptor (r) 1 was localized to FSH cells and detected only in juvenile fish, while GnRrhr3 was found in LH cells across both juvenile and mature stages. GnRH stimulation significantly increased LH release, while FSH levels showed only a moderate rise. Structural analysis revealed that Gnrhr3 exhibits more stable and rapid ligand binding and activates protein kinase A and protein kinase C (PKC) pathways, whereas Gnrhr1 signals exclusively through the PKC/Ca^2+^ pathway. Elevated FSH levels and *gnrhr* *1* expression were observed during early vitellogenesis, coinciding with increased *cck-rba* expression in FSH cells. Cck-positive neurons originating in the brain were observed to terminate in the adenohypophysis, specifically near FSH-secreting cells. In addition, *c* *ck-rba* was exclusively expressed in FSH cells, and tilapia Cck injection significantly elevated plasma FSH levels while reducing food intake, highlighting the role of Cck in linking reproduction to nutritional status. Together, these findings indicate that Gnrh predominantly regulates LH secretion via Gnrhr3, while Cck, through Cck-rba, specifically controls FSH, linking reproduction to nutritional status. Cck acts as a metabolic gatekeeper, aligning gonadal development with energy availability and ensuring reproduction when energy is sufficient.

## Introduction

The endocrine system regulates numerous physiological processes in vertebrates, including reproduction and somatic growth. Reproduction is controlled by the hypothalamus–pituitary–gonad (HPG) axis, which responds to environmental and endogenous cues such as photoperiod, temperature, behavior, pheromones, and nutritional status changes. In fish, gonadal growth and maturation are regulated by two distinct gonadotropins: follicle-stimulating hormone (FSH), which stimulates the maturation of follicles in females and sperm production in males, and luteinizing hormone (LH), which triggers final gonadal maturation in males and females (Levavi-Sivan *et al.* 2010). Unlike mammals, in fish, gonadotropins are secreted from dedicated cells in the pituitary with a unique morphology (Hollander-Cohen *et al.* 2021).

Gonadotropin-releasing hormone (Gnrh), a neuropeptide hormone, plays a crucial role in reproductive events by controlling the release of gonadotropins from the anterior pituitary gland in all vertebrates (Millar 2005). Three forms of GnRH are present in early- and late-evolving bony fishes: chicken GnRH-II (GnRH2) is expressed by midbrain neurons, a species-specific GnRH (Gnrh1) is the hypophysiotropic form that is present in the preoptic area and hypothalamus, and salmon GnRH (Gnrh3) is localized in the terminal nerve ganglion (Somoza *et al.* 2002). The diversity and paralogs of GnRHRs in vertebrates result from a complex evolutionary history involving local gene duplications, whole-genome duplication events, and lineage-specific adaptations (Millar 2005). This diversity is also the reason for the complicated nomenclature of these peptides and their receptors. GnRH receptors on the pituitary gonadotrophs mediate the effects of GnRH, ultimately influencing FSH and LH secretion (Levavi-Sivan *et al.* 2010). The hypothalamic-LH pathway is the most extensively studied of the two gonadotropins, whereas the hypothalamic-FSH pathway has been less studied in vertebrates, including teleosts (Levavi-Sivan *et al.* 2010, Molés *et al.* 2020, Biran *et al.* 2024).

Our recent RNA-seq findings revealed that FSH and LH cells express different paralogs of the GnRH receptor and that FSH cells show high cholecystokinin (CCK) receptor-ba (*cck-rba*) expression (Hollander-Cohen *et al.* 2021). CCK peptide is an essential pancreatic secretion factor in the gastrointestinal tract. It is well established that CCK has numerous functions in mammals: it controls the secretion of enzymes, gallbladder contraction, intestinal motility, and satiety, and inhibits gastric acid secretion (Moran 2000). In addition, CCK regulates brain dopamine, anxiety, panic, and locomotion (Moran & Schwartz 1994). There are two CCK-R subtypes. CCK-RA is predominantly expressed in the gastrointestinal system, whereas CCK-RB is expressed in the central nervous system, where it significantly affects satiety, memory, anxiety, osmotic stress, and neuropsychiatric disorders (Wank 1995). We recently found that teleosts possess a distinct type of Cck-r, previously designated as Cck-r-like, as it lacks a mammalian ortholog. We showed that this receptor binds and is activated by CCK, so we termed this receptor Cck-rba. Cck-rba is the sole CCK receptor expressed in the pituitary gland. Several studies have demonstrated its critical role in regulating FSH secretion in zebrafish and medaka, where the knockout of Cck-rba significantly disrupts FSH secretion and gonadal development (Hollander-Cohen *et al.* 2021, Hollander-Cohen *et al.* 2024, Uehara *et al.* 2024). This highlights its unique function in bridging the somatic and gonadal axes.

GnRH receptors (GnRHr) are members of the rhodopsin β subfamily of the G protein-coupled receptors (GPCRs) that specifically interact with all GnRH peptides. There was a notable duplication pattern and loss of specific GnRHR types across vertebrate groups. Gene duplication followed by functional divergence generates hormone subtypes and their cognate receptors. This has resulted in using various nomenclature systems for the GnRHR subtypes. Among the multiple nomenclature systems proposed for GnRHR subtypes, we adopted the classification suggested by (Levavi-Sivan & Avitan 2005, Millar 2005), which identified three distinct classes of GnRHRs (Types I, II, and III), further subdivided into five classes (Andersson *et al.* 2023). The number of GnRHRs varies among fish species: five Gnrhr types have been found in European sea bass (*Dicentrarchus labrax*) (Moncaut *et al.* 2005) and pufferfish *Tetraodon nigroviridis* (Ikemoto & Park 2005), and four in zebrafish (Tello *et al.* 2008) and grass carp (Li *et al.* 2022).

It is well established that growth and reproduction are tightly linked in all animals, and in fish, a shift in energy utilization occurs between the somatic and gonadal axes during the reproductive season. The somatic axis initially dominates during growth, while the gonadal axis is weaker and gradually gains prominence as the organism transitions to reproductive maturity, reflecting shifting physiological priorities during the life cycle. Understanding and utilizing these processes are crucial for the successful cultivation of animals. Previous RNA-seq findings indicated that FSH and LH cells express different GnRH receptor paralogs and that FSH cells show high CCK receptor expression, suggesting a more complex regulatory interplay. Building upon these insights, we hypothesized that Gnrh and Cck would differentially regulate FSH and LH secretion in tilapia, thereby revealing distinct hypothalamic pathways associated with different stages of reproduction and metabolic status.

This study used tilapia to investigate the differential regulation of Gnrh and Cck during reproduction and feeding. Here, we demonstrated that the two types of Gnrh receptors expressed on FSH and LH cells differ in their localization within the pituitary cells, signal transduction pathways, expression patterns during sexual development, structural homology, and functional activation. These differences highlight different regulatory pathways in the fish pituitary. Moreover, our findings revealed that the satiety hormone Cck functions as a significant neuropeptide in Nile tilapia, an important commercial fish species. Identifying the role of Cck in FSH modulation adds an essential layer of understanding to the intricate hormonal control of reproductive processes in fish.

## Materials and methods

### Fish maintenance and growth

Nile tilapia (*Oreochromis niloticus*) were housed and bred in a fish facility unit at the Hebrew University in 500-L tanks maintained at 26°C with a 14L/10D photoperiod. The fish were fed commercial fish pellets (Raanan fish feed, Israel) containing 6% fat and 50% protein *ad libitum*. All *in vivo* experiments were conducted on sexually mature fish. For *in situ* hybridization chain reaction (HCR), both mature and juvenile fish were sampled. We followed the Animal Care and Use guidelines of Hebrew University, and the study was approved by the local Administrative Panel on Laboratory Animal Care (ethics approval Nos. AG21-16615 and AG17-15120).

### *In situ* HCR, clearing, and immunofluorescence on double-labeled pituitary

Transgenic tilapia expressing GFP in FSH cells and RFP in LH cells (Golan & Levavi-Sivan 2013, Golan *et al.* 2014) were used for HCR and immunofluorescence assays, according to (Mizrahi *et al.* 2024). Tilapia pituitaries were collected from juvenile and mature female fish (BW, 35 ± 5.60 g and 74.15 ± 12.03 g, respectively), with gonadosomatic index (GSI; the ratio of fish gonad weight to their BW X 100) values of 0.19 ± 0.05% and 3.57 ± 1.34%, respectively. Briefly, pituitaries were fixed in 4% paraformaldehyde in PBS for 6 h at 4°C and then immersed in PBS containing 20% sucrose and 30% OCT (Sakura) for 24 h. The glands were embedded in OCT, frozen, sectioned, and mounted on Superfrost Plus glass slides (Thermo Fisher Scientific, USA). Samples were stored at −80°C. Single-molecule RNA HCR was performed following the HCR protocol adapted from Molecular Instruments HCR v3.0, as described by (Choi *et al.* 2018). Frozen sections were thawed, fixed in 4% paraformaldehyde in PBS for 15 min at 4°C, and immersed in ethanol (50, 70, 100%) for 5 min each. Sections were incubated with proteinase K and prehybridized in the probe hybridization buffer for 10 min at 37°C. Slides were then incubated with 0.4–0.8 pmol of denaturation probes (designed specifically for each tilapia Gnrhr (mature and juvenile fish) or Cck-rba (mature fish) by molecular instruments). After hybridization, the sections were washed in decreasing concentrations of probe wash buffer and finally in 5xSSCT solution. The slides were preamplified in an amplification buffer and incubated overnight in a dark chamber with snap-cooled h1 and h2 hairpins. Excess hairpins were removed using three 5xSSCT washes. The slides were stained with DAPI and mounted in an anti-fade solution. The pituitaries were imaged using a confocal fluorescent microscope (Leica Microsystems, Germany), and images were processed using the Fiji program (Schindelin *et al.* 2012).

The pituitary glands of sexually mature adult tilapia were cleared using the CUBIC method, according to (Susaki *et al.* 2015). Briefly, the pituitary was fixed by immersion in PFA at 4°C for 18 h and washed of the remaining PFA with PBS (0.01% w/v sodium azide). Next, the pituitary was immersed in half-diluted reagent 1 (urea (25% wt/vol), Quadrol (25% wt/vol), Triton X-100 (15% wt/vol) in dH2O) at 37°C overnight, then the medium was switched to complete reagent 1. The immunostaining protocol was initiated when the pituitary was cleared with reagent 1. The cleared pituitary was incubated with rabbit anti-cholecystokinin (CCK-8; Merck, Istrael; 1:1,000) for 16 h at 37°C, washed, and incubated with goat anti-rabbit. This antibody has previously been validated for use in several fish species, including zebrafish (Hollander-Cohen *et al.* 2024), goldfish (Himick & Peter 1994), and red drum (Webb *et al.* 2010). The tissue was washed with PBS/0.01% (w/v) sodium azide and stained with DAPI. After staining the tissue, the CUBIC protocol was continued. The tissue was incubated in half-diluted reagent 2 (urea (25% wt/vol), sucrose (50% wt/vol), and triethanolamine (10% wt/vol) in dH2O) at 37°C overnight, then the medium was switched to complete reagent 2.

### Luciferase reporter gene assay

To investigate the signaling pathways of different tilapia Gnrh receptors and the Cck receptor, precisely to distinguish between the different signal transduction pathways, we employed a sensitive luciferase (LUC) reporter gene assay (Cheng *et al.* 2010). Luciferase (luc) transactivation assays have been validated to discriminate different GPCR pathways, such as cAMP response element (CRE-luc), serum response element (SRE-luc), and nuclear factor of activated T-cells response element (NFAT-RE-luc) for adenylate cyclase (AC)/cyclic adenosine monophosphate (cAMP)/protein kinase A (PKA), extracellular signal-regulated kinase (ERK)/mitogen-activated protein kinase (MAPK), and intracellular Ca^2+^ mobilization, respectively. Receptor activation was performed as described previously (Biran *et al.* 2014*a*, *b*, Mizrahi *et al.* 2019, 2024). EC50 values were derived from concentration–response curves using computerized nonlinear curve fitting in Prism version 10 software (GraphPad). Four experiments were conducted using distinct batches of COS-7 cells, with three replicates per concentration. The data presented are the averages of four experiments.

### Effect of GnRH or tilapia CCK on gonadotropin release *in vivo*

Over the years, numerous *in vivo* experiments have been performed using various secretagogues, including neurokinin (NK) NKB/NKF, Spexin, dopamine, and somatostatin (Biran *et al.* 2014*b*, Mizrahi *et al.* 2019, 2024, Cohen *et al.* 2020, Mizrahi *et al.* 2024). Adult female tilapia (92.05 ± 12.01 BW; mean ± SEM) were injected with either sGnRHa (10 μg/kg BW; [D-Ala^6^, Pro^9^-Net]-mammalian GnRH; GL Biochem, China) as a positive control or fish saline as a negative control. All trials were conducted within the same water tanks and at consistent times of the day to control for environmental variables. We initially calculated the mean hormone concentrations of the baseline samples (time zero) for FSH and LH to normalize the data. Each subsequent sample was then normalized by dividing it by the baseline average, allowing for a standardized comparison of hormone concentration changes over time within and across treatment groups.

Female tilapia exhibit asynchronous oocyte development and batch spawning patterns, releasing eggs every 2–3 weeks. Consequently, a group of females represents all reproductive cycle stages at any given time. Therefore, by sampling fish at a specific time, we captured all stages of the female tilapia reproductive cycle. Fish were divided into four specific reproductive groups according to their gonadosomatic index (GSI) and follicle diameter: primary follicles (PF), early vitellogenic (EV), vitellogenic (V), and mature follicles (MF). Blood was sampled from the caudal blood vessels, the pituitaries were collected for RT-PCR, and histological analysis was performed according to (Hurvitz *et al.* 2005, Jackson *et al.* 2006).

For analysis of the effect of tilapia (ti) Cck on gonadotropin release, a total of 80 adult male Nile tilapia (BW 72.01 ± 9.71 g; 16 fish/group) were injected intraperitoneally (IP) with saline and tiCck (D-Y[SO3H]-L-G-W-M-D-F-NH_2_; GL Biochem, China) at concentrations ranging from 10 to 500 μg/kg. Fish were bled from the caudal blood vessels into heparinized syringes at 2, 4, 8, and 24 h after injection. Blood was centrifuged at 3,000 ***g*** for 30 min at 4°C to obtain plasma samples, which were then stored at −20°C until assayed.

### Quantification of food intake

A total of 24 adult male tilapia (BW 116.16 ± 15.07 g; mean ± SEM) were fed daily for 2 weeks before the beginning of the experiment. To quantify the daily food intake, fish were overfed once daily at 09:00 h, offering one pellet at a time until satiation was reached. More than 4% BW ratio of floating pellets per fish was administered 1 h post IP tilapia Cck injection (0, 10, or 100 μg/kg BW; 2 tanks with 4 fish/treatment; *n* = 8 fish/group; 6 tanks total). Since Cck is a postprandial short-term satiety factor in mammals and fish (Ronnestad *et al.* 2017), food consumption was measured during the first 3 h after injection. Food was weighed in 2 g portions in test tubes and gradually fed. Fish were classified as satiated if all the fish in a group rejected three pellets in a row for 1 min. Food consumption was converted to grams of food consumed based on the mean pellet weight.

### ELISAs for the measurement of tilapia FSH and LH

A specific homologous competitive ELISA based on recombinant (r) tilapia (t) gonadotropins (GTHs) was used to measure FSH and LH levels in the plasma developed for tilapia (Aizen *et al.* 2007*b*). Antibodies were produced against rtFSHβ and rtLHβ, and rtFSHßα and rtLHßα were used to generate a standard curve (Kasuto & Levavi-Sivan 2005, Aizen *et al.* 2007*a*). The sensitivities of plasma measurements were 15.84 pg/mL for LH and 0.24 pg/mL for FSH. The interassay coefficients of variation (CV) were 14.8 and 12.5%, whereas the intraassay CVs were 7.2 and 8% for LH and FSH, respectively.

### RNA extraction and real-time PCR

Pituitaries were harvested at the end of the *in vivo* experiment after 24 h, snap-frozen in liquid nitrogen, and stored at −80°C. Total RNA was extracted using phenol-chloroform with Trizol (Ambion, USA) according to the manufacturer’s protocol. An additional cleaning step was performed, which included DNase I treatment (RNA Clean & Concentrator kit, Zymo Research, USA). The RNA was then quantified using a NanoDrop spectrophotometer (Thermo Fisher), and integrity verified by agarose gel electrophoresis. cDNA was synthesized from 1 μg of total RNA using the Verso cDNA kit (Thermo Scientific, USA), following the manufacturer’s protocol with oligo (dT) and random hexamer primers, and with no-reverse transcriptase (–RT) controls to assess genomic DNA contamination. Primer pairs were designed to span exon–exon junctions when possible. For each primer pair, a standard curve was generated using a serial dilution of pooled cDNA to determine assay efficiency and *R* ^2^ values (Table 1S (see section on Supplementary materials given at the end of the article)). Primer specificity was confirmed by melt-curve analysis, and efficiency was determined from standard curves. qPCR was performed on a real-time PCR instrument (Roche LightCycler 96) using SYBR Green Master Mix with technical replicates and no-template controls for each reaction. The PCR mixture consisted of 3 μL of diluted cDNA sample (1:3), 200 μM of each primer, and 10 μL of SYBR Green qPCR SyGreen Blue Mix (PCR Biosystems, UK) in a final volume of 20 μL. The thermal cycling program for SYBR Green assays was: initial activation at 95°C for 120 min, followed by 40 cycles of 95°C for 15 s and 60°C for 30 s (anneal/extend); a melt curve was performed from 65 to 97°C with 0.2°C increments, all in a 96-well plate. The specificity of the amplification was tested at the end of the PCR by melting-curve analysis and sequencing the PCR products. Target gene expression was normalized to the geometric mean of two validated reference genes (housekeeping) that were shown to be suitable for both tissue analysis and developmental time studies of fish (β actin and elongation factor 1α (*ef1α*) (Tang *et al.* 2007)), selected based on stability testing, and relative expression calculated using the 2^–ΔΔCt method. The study was conducted in full accordance with the Minimum Information for Publication of Quantitative Real-Time PCR Experiments (MIQE) guidelines (Bustin *et al.* 2009).

### Homology model and binding site of Gnrhrs

An *in* *silico* model for tilapia Gnrhr1 (NCBI accession no. NP_001266689) and Gnrhr3 (NCBI accession no. XP_019215598) was generated using the I-TASSER server (Zhang 2008, Roy *et al.* 2010), based on the huGnRH1R model 7BR3 (Yan *et al.* 2020) as a template structure. The models were further prepared, and binding sites were predicted using Schrödinger (BioLuminate, Schrödinger, LLC, USA, 2020), according to Atre *et al.* (2024).

### Statistical analysis

The results are presented as the mean ± SEM. Two-way ANOVA and one-way ANOVA were used for ELISA and qPCR, respectively, to compare the mean values of the *in vivo* experiments. A posteriori Tukey-Kramer test (all pairs) was used only when ANOVA revealed statistically significant differences between groups using JMP Version 11 software. Four independent experiments were performed for receptor transactivation assays, each independently and in triplicate. The EC50 values were calculated from the concentration–response curves using computerized nonlinear curve fitting for all experiments. Statistical significance was set at *P* < 0.05 level.

## Results

### Differential localization and temporal expression of Gnrh receptor types in gonadotropin cells of the pituitary

In the current study, we focused on two forms of Gnrhr found by RNA-seq to be expressed in the tilapia pituitary, which we named Gnrhr1 and Gnrhr3 (Levavi-Sivan *et al.* 2005, Hollander-Cohen *et al.* 2021). The tilapia Gnrhr1, which was found to be expressed in FSH cells (Hollander-Cohen *et al.* 2021), is similar, in terms of sequence homology, to Gnrhr1 in the Atlantic cod (von Krogh *et al.* 2017), Gnrhr1b in medaka (Strandabø *et al.* 2013), Gnrhr1cb in lumpfish (Andersson *et al.* 2023), and Gnrhr2 in Astatotilapia (Chen & Fernald 2008). The tilapia Gnrhr3, which is expressed in LH cells (Hollander-Cohen *et al.* 2021), is similar to the Gnrhr2 in Atlantic cod (von Krogh *et al.* 2017), Gnrhr2a in medaka (Strandabø *et al.* 2013), Gnrhr2ba1 in lumpfish (Andersson *et al.* 2023), and Gnrhr1 in Astatotilapia (Chen & Fernald 2008).

**Figure 1:**
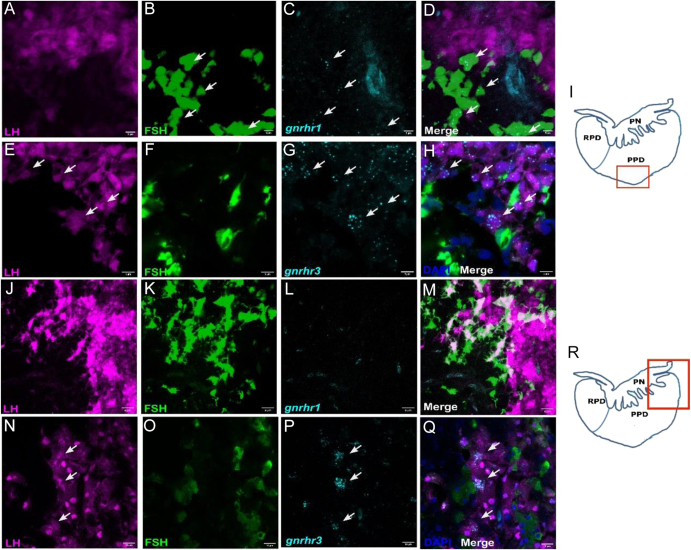
*gnrh* *r1* and *gnrh* *r3* mRNA localization in juvenile or mature tilapia pituitary using *in situ* HCR. Double-labeled pituitaries of transgenic fish expressing LH (A, E, J, N) and FSH (B, F, K, O) cells with specific fluorophores were used for cellular co-localization of *gnrh* *r1* (C and L) and *gnrh* *r3* (,G and, P) in juvenile fish (A, B, C, D, E, F, G, H) or mature fish (J, K, L, M, N, O, P, Q). Scale bar, 10 μm. Drawings on the right (I and R) mark the location of the imaged area within the pituitary.

To investigate the cellular basis of differential gonadotropin regulation, we hypothesized that distinct types of Gnrh receptors would be precisely and differentially localized within specific gonadotropin cell types. Our primary objective was to precisely localize the different types of Gnrh receptors in various gonadotropin cell types using *in situ* HCR with specific probes for each receptor. To confirm the expression of fluorophores in the proximal pars distalis (PPD), we utilized double-labeled pituitary samples from fish expressing RFP in LH cells (Fig. 1A, E, J, N) and GFP in FSH cells (Fig. 1B, F, K, O) (Golan & Levavi-Sivan 2013, Golan *et al.* 2014). Each fluorophore was exclusively expressed in different cell types, with FSH cells located dorsal to the LH cells, closer to the dorsal projections of the pars nervosa. We found that each receptor had cell-specific temporal expression; *gnrhr1* expression was detected only in the pituitary of juvenile fish (Fig. 1C and D), whereas *gnrhr3* was expressed in both juvenile (Fig. 1G and H) and mature fish (Fig. 1P and Q). In addition, *gnrhr*1 predominantly co-localized with FSH cells of juvenile fish (Fig. 1C and D) but not with those of mature fish (Fig. 1L and M), whereas *gnrhr3* was expressed in the LH cells of juvenile (Fig. 1G and H) and mature fish (Fig. 1P and Q). The anatomical context of the imaged regions within the pituitary is illustrated in schematic drawings (Fig. 1I and R). These findings demonstrate developmental stage–dependent expression of *gnrh* *r1* and *gnrh* *r3*, with preferential localization to FSH and LH cells, respectively.

### The differential effect of Gnrh on the release of FSH and LH

**Figure 2:**
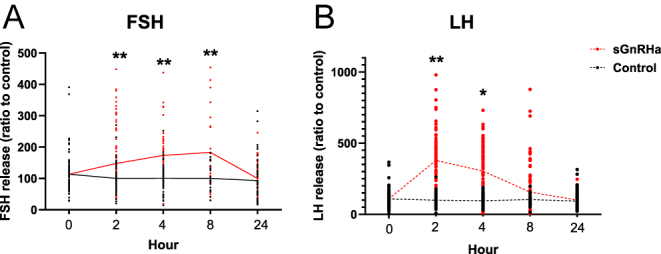
*In vivo* effect of sGnrha on GTH release. Mature tilapia males were intraperitoneally injected with sGnrha (10 μg/kg body weight (BW)) and saline as a control. Blood was sampled at 0, 2, 4, 8 and 24 h post-injection. Plasma FSH (A) and LH (B) values were analyzed by specific ELISAs (mean ± SEM; at 0, 2, 4, 8, and 24 h: FSH, control: *n* = 82, 78, 62, 58, 58; sGnRHa: *n* = 82, 82, 70, 58, 58. LH, control: *n* = 82, 71, 58, 58, 58; sGnrha: *n* = 82, 78, 78, 62, 58 fish/group). Columns marked by asterisks significantly differ from time 0: *P < 0.05; **P < 0.01.

To evaluate Gnrh’s effect on LH and FSH release into the plasma, sexually mature female tilapia were administered an intraperitoneal injection of sGnrha (10 μg/kg BW). Plasma gonadotropin levels were quantified using specific ELISAs at 2, 4, 8, and 24 h post-injection, as well as in control fish, to provide baseline measurements (Fig. 2). To evaluate the specific impact of Gnrh, we hypothesized that Gnrh would primarily elicit a significant increase in LH levels, with a more moderate or limited effect on FSH release in sexually mature female tilapia. Gnrh treatment elicited a significant increase in LH levels compared to FSH levels at 2 and 4 h post-injection relative to the control fish (Fig. 2B). In contrast, although statistically significant, the FSH levels exhibited a more moderate elevation than those of the control (Fig. 2A).

### Morphological, hormonal, and gene expression changes during reproductive development in female tilapia

**Figure 3:**
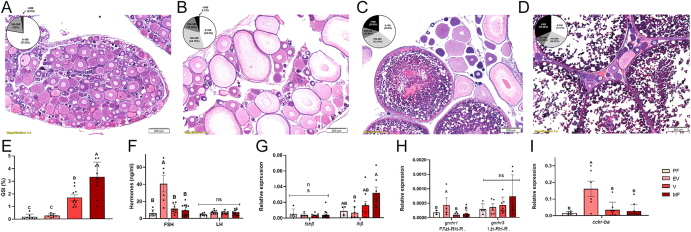
Comparison of different reproductive parameters in females at specific stages of ovarian development. Representative histological characteristics of the ovary at various developmental stages (A, B, C, D). Oocyte size frequency distribution between the different stages is presented in pie charts (A, B, C, D). Primary follicle (PF, A); early vitellogenic (EV, B); vitellogenic (V, C); mature follicles (MF, D). Gonadosomatic index (GSI, E); FSH and LH plasma levels (F); pituitary *fshβ *and *lhβ *(G); *gnrhr1* and *gnrhr3* (H); and *cck-* *rba* (I) mRNA levels. The results are mean ± SEM (*n* = 5–12; Table S4). Means marked with different letters are statistically different.

Next, we aimed to identify the reproductive profile of the tilapia pituitary throughout sexual development in terms of hormone release, gonadal histology, and gene transcripts. Because tilapia are batch-spawning fish with ovaries containing follicles at various reproductive stages (Coward & Bromage 2000), the maturation stage of each fish was determined according to their GSI and follicle diameter. The primary follicle stage mainly comprised prenuclear follicles with large central nuclei (Fig. 3A). The early vitellogenic stage contained larger follicles with cortical alveoli in their cytoplasm and large follicles with oil vacuoles and yolk granules accumulated in the cytoplasm (Fig. 3B). The vitellogenic stage contained follicles in which the yolk granules became yolk plates (Fig. 3C). The mature stage contained follicles where the germinal vesicle started to migrate to the periphery and went through the dehydration stage, where the follicle reached its maximal size (Fig. 3D). As expected, as indicated by the GSI, ovarian growth increased in parallel with the ovarian stage, peaking in the group with mature follicles (Fig. 3E). Plasma FSH levels were significantly higher during the early vitellogenic stages. In contrast, LH levels showed no significant differences across ovarian stages (Fig. 3F). Regarding gene expression, *fshß* levels showed no significant differences (Fig. 3G), coinciding with a significant increase in *cck-rba* levels (Fig. 3I) and FSH release (Fig. 3F). Although *gnrh* *r3* expression increased as the gonads developed, this increase was not statistically significant (Fig. 3H).

### Gnrh receptors have different structural properties and signaling pathways

**Figure 4:**
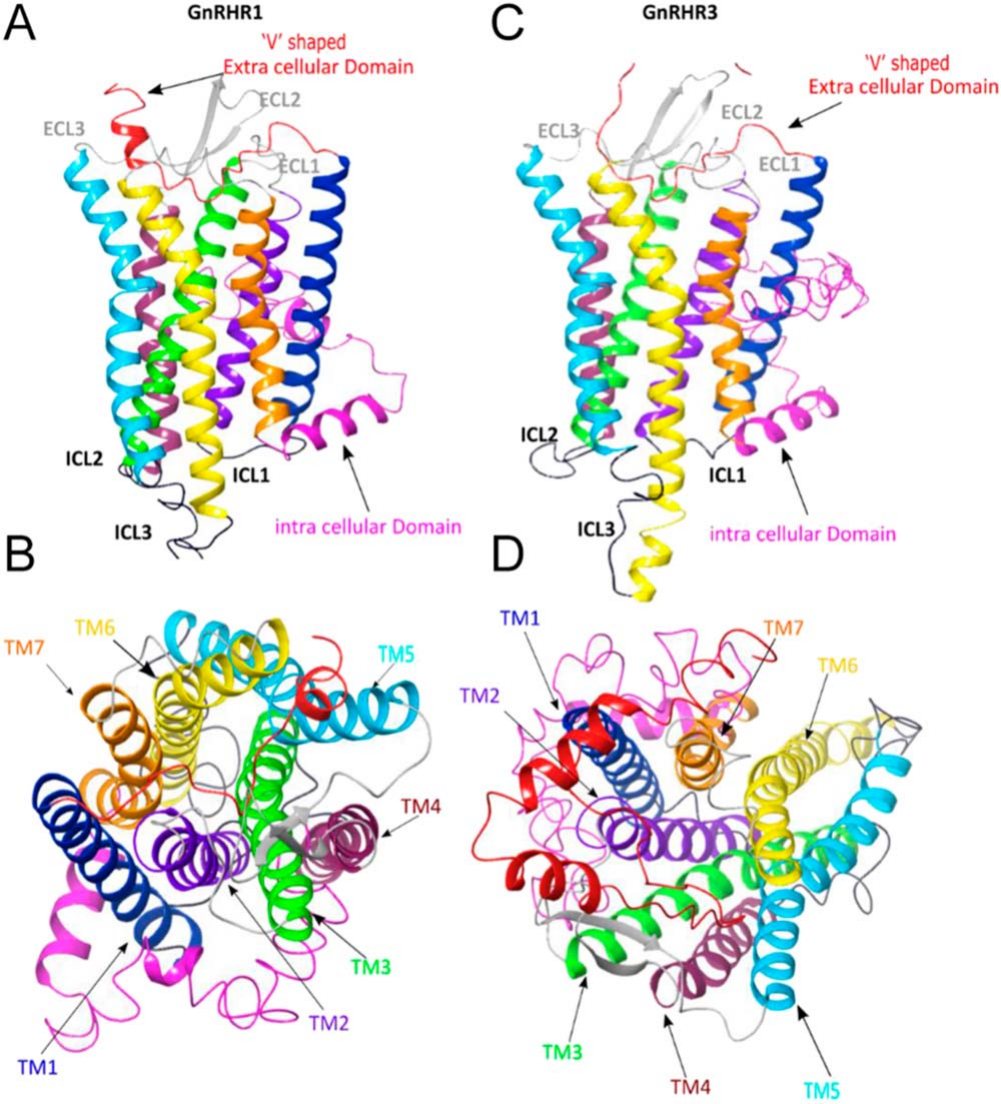
Homology models. Predicted model of tilapia Gnrhr1 (top view: A; side view: B) and tiGnrhr3 (top view: C; side view: D) based on C-score prediction, generated using I-TASSER. The domains of GPCR are expressed as follows: exhibiting ‘V’-shaped extracellular domain (red), seven transmembrane domains (TM1-blue; TM2-indigo; TM3-green; TM4-purple; TM5-aqua blue; TM6-yellow; TM7-orange), and a C-terminal domain (pink), extracellular loops (gray), and the intracellular loops (black).

To identify differences in the structure that reflect the binding capacity to the ligand, we reconstructed each receptor using homology models for tiGnrhr1 and tiGnrhr3 generated on the I-TASSER server (Zhang 2008, Roy *et al.* 2010, Yang *et al.* 2015) using the huGnRH1R structure 7BR3 (Yan *et al.* 2020) as a template. Distance errors were estimated using support vector regression utilizing the convergence of threading alignment, sequence-based secondary structure, and solvent accessibility predictions. The models were selected based on structural stability, template similarity, and high confidence score (C-score). The receptor structures were observed to belong to the β-branch of class A (Rhodopsin) GPCRs. Unlike huGnRH1R, but similar to other piscine Gnrhrs, both tilapia Gnrh-rs consist of a cytoplasmic C-terminal helix (helix 8). The binding pockets are localized within the transmembrane domains of both tilapia Gnrhrs. Given that GnRH2 is the most conserved form of GnRH and is present in all vertebrates (Desaulniers *et al.* 2017), we analyzed its docking conformation and binding interactions with Gnrhr1 and Gnrhr3 (Fig. 4A and B). Interestingly, Gnrh2 induces different activation patterns for each receptor. The binding pocket for tiGnrhr3 was observed to possess a more horizontal conformation, similar to that of huGnRH1R, whereas tiGnrhr1 was predicted to have a narrower binding pocket with a much deeper and more vertical hydrophobic cleft (Fig. 4C and D; Supplementary Table S2). Structural analysis revealed that Gnrhr3 binding to the ligand is more stable than Gnrhr1.

**Figure 5:**
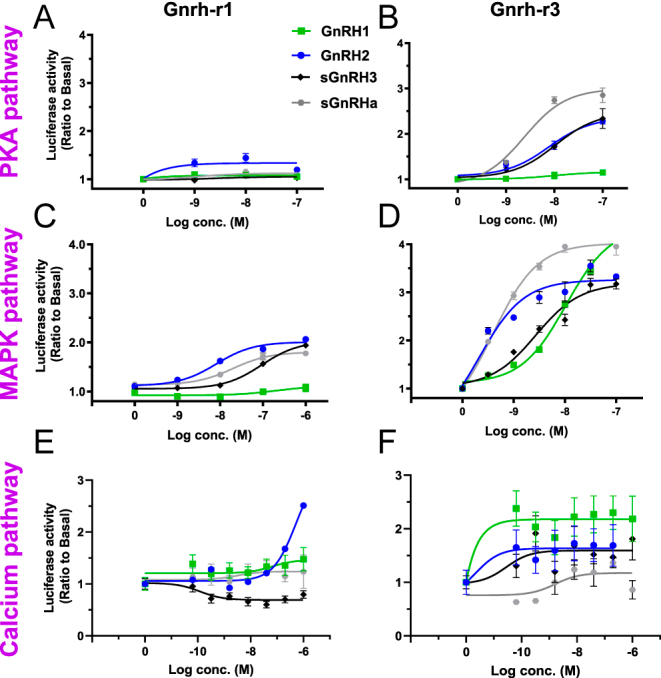
Signal transduction pathways of the different Gnrhr types. COS-7 cells were transfected with tilapia Gnrhr1 (A, C, E) and Gnrhr3 (B, D, F) along with cAMP response element (CRE)-luc (A and B), serum response element (SRE)-luc (C and D), or nuclear factor of activated T cells (NFAT)-luc (E and F). The cells were treated with various concentrations of Gnrh1 (sea bream Gnrh), GnRH2 (chicken GnRHII), GnRH3 (salmon Gnrh), and salmon Gnrh analog (sGnrha). The data are expressed as the change in luciferase activity over basal activity. Each point was determined in triplicate and is presented as a mean ± SEM.

Although the same ligand activates both Gnrh receptors, they exhibit differences in the signaling pathways and structures that underlie the differential regulation of Gnrh on LH and FSH. We investigated the specific intracellular signaling of each Gnrhr type using COS-7 cells transfected with tilapia Gnrhr1 or Gnrhr3 together with specific reporter plasmids for the cAMP/PKA, ERK/MAPK or Ca^2+^/PKC pathways (CRE-Luc, SRE-Luc and calcium mobilization; (NFAT-Luc), respectively) (Biran *et al.* 2008, Cheng *et al.* 2010). Notably, differences were observed in terms of minimal effective dose, maximal responses, and signal-transduction pathways of the two Gnrhr types. Gnrh-r1 (expressed in FSH cells) relayed its activity only through the ERK/MAPK and PKC/Ca^2+^ pathway, with relatively low maximal responses and high EC50s (Fig. 5A, C, E; Supplementary Table S3). However, Gnrhr3 (expressed in LH cells) mediated its intracellular signaling through the PKA, MAPK, and calcium pathways, with high maximal responses and low EC50s (Fig. 5B, D, F; Supplementary Table S3). Gnrhr3 exhibited more efficient activation via the Ca^2+^/PKC pathway compared to the cAMP/PKA pathway. All Gnrh forms activated both receptors with different efficiencies, minimal effective doses, and maximal responses. This confirms that the major downstream signaling pathways for Gnrhr3 are via Gq and Gs proteins, whereas those for Gnrhr1 are only via Gq protein.

### Cck-rba co-localizes with FSH cells and activates the PKA/cAMP pathway

**Figure 6:**
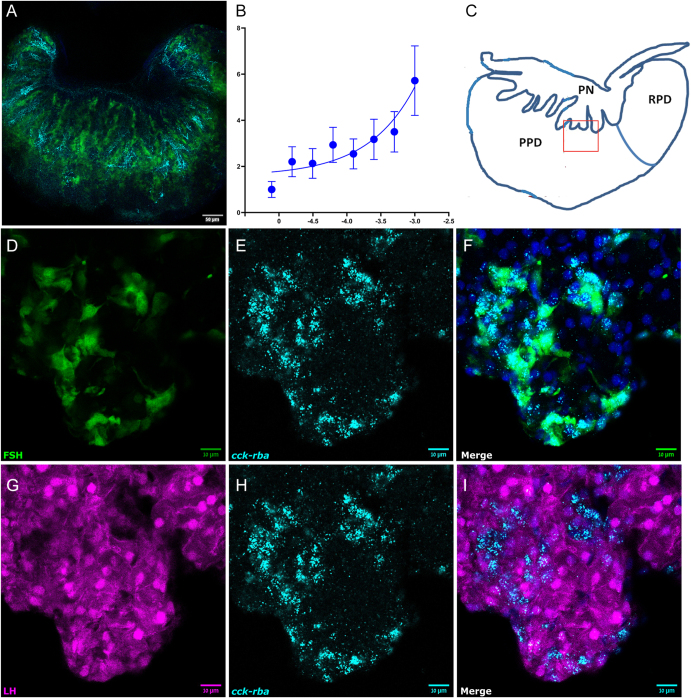
Receptor activation and localization of Cck and Cck-rba in tilapia pituitary. The whole tilapia pituitary was cleared by the CUBIC method and stained with antibodies against the CCK peptide. FSH cells are shown in green, and the CCK peptide is shown in cyan; scale bar, 50 μm (A). Signal transduction pathways of the Cck-rba. The data are expressed as the change in luciferase activity over basal activity. Each point was determined in triplicate and is presented as a mean ± SEM (B). The whole pituitary box indicated the area enlarged in images D–I (C). mRNA expression analysis of *cck-* *rba* in tilapia pituitary. Double-labeled pituitaries from transgenic fish with labeled FSH cells (green; D) and LH cells (magenta; G) were used for *in situ* HCR to determine co-localization of mRNA expression of *cck–* *rba* (cyan; E and H). Nuclei are stained with DAPI. In the merged box, green shows FSH (F), and magenta shows LH cells (I). Scale bar, 10 μm.

To determine whether CCK-expressing neurons extend to the pituitary, mainly targeting FSH-secreting cells, we applied a pituitary clearing technique followed by immunofluorescence analysis. This approach allowed us to trace CCK-positive neurons originating in the brain, which were observed to terminate in the adenohypophysis, specifically near FSH-secreting cells (Fig. 6A). This finding supports the potential role of CCK in the modulation of FSH activity in the pituitary gland. We also aimed to confirm a specific signaling pathway activated by tilapia Cck through its receptor. Tilapia Cck dose-dependently activates tilapia Cck-rba and transduces its activity through the PKA/cAMP pathway (Fig. 6B and C).

Based on our previous RNA-seq data showing extremely high expression of *cck-rba* in FSH cells (Hollander-Cohen *et al.* 2021), we hypothesized that CCK-expressing neurons would terminate specifically near FSH-secreting cells, and that the Cck-rba would exclusively co-localize with FSH cells in the pituitary. We used double-labeled tilapia, in which FSH cells were marked with GFP and LH cells were marked with RFP (Fig. 6D and G). When comparing the co-localization of the receptor on FSH and LH cells, there was an explicit expression of *cck-rba* on FSH cells (Fig. 6D, E, F) and no expression on LH cells (Fig. 6, G, H, I).

### Effect of tilapia CCK on LH and FSH release, synthesis, and feeding rate

**Figure 7:**
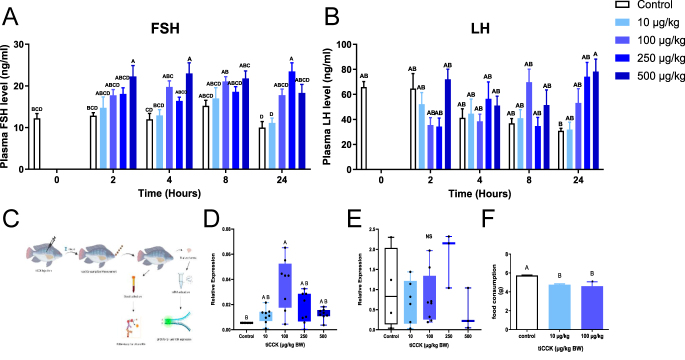
Effect of tilapia Cck intraperitoneal injection on LH and FSH release and gene expression *in vivo*, and food intake. Male tilapia were intraperitoneally injected with tilapia (ti) Cck peptide at elevated concentrations (10, 100, 250, 500 μg/kg body weight (BW)), and the control group was injected with fish saline. Blood was sampled at 0, 2, 4, 8, and 24 h post-injection (C). FSH (A) and LH (B) plasma levels are shown as mean ± SEM (*n* = 16 fish/group). Pituitary mRNA levels of *fshβ *(D) and *lhβ *(E) were determined by real-time PCR (mean ± SEM; *n* = 8 fish/group). The effect of tiCck on daily food intake is shown in (F) (mean ± SEM; *n* = 8 fish/group). NS, non-significant. Different letters denote significant differences; *P* < 0.05. (Panel C was created using BioRender.com).

To further investigate the physiological role of CCK in gonadotropin release, and its link to metabolism, we hypothesized that tilapia Cck injection would selectively elevate plasma FSH levels and *fshb* mRNA expression, with minimal or no significant effect on LH secretion or lhb expression, and would concomitantly reduce food consumption, thereby demonstrating its role as a metabolic gatekeeper. We intraperitoneally injected elevated concentrations of tilapia Cck and bled the fish at 0, 2, 4, 8, and 24 h post-injection (Fig. 7C). As fast as two hours post-injection, there was a significant increase in plasma FSH levels with the treatment of 100, 250, or 500 μg/kg BW. A significant increase was observed at 4 and 8 h after injection in the 100 and 500 μg/kg BW treatments. At 24 h post-injection, a significant increase in FSH secretion was observed with 100, 250, and 500 μg/kg BW (Fig. 7A). However, LH secretion in response to the tilapia (ti) Cck injections was mixed. Reduced plasma levels at 2 h post-injection were observed at concentrations of 100 and 250 μg/kg BW; no significant change in plasma LH levels was observed at 4 h post-injection. Twenty-four hours post-injection, plasma LH levels were significantly increased at the highest concentrations (Fig. 7B). *fshß* mRNA expression levels gradually increased following tiCck injection, peaking at 100 μg/kg BW, before decreasing (Fig. 7D), while no significant gene expression was evident in *lhß* (Fig. 7E). tiCck significantly reduced food consumption during the first 3 h after the beginning of feeding by more than 10% at 10 and 100 μg/kg BW (Fig. 7F).

## Discussion

While the HPG axis is a critical regulator of reproduction and has been widely investigated in mammals and fish, a significant gap exists in understanding the distinct pathways leading to the synthesis and release of FSH compared with LH in all vertebrates, despite this. This study directly addresses this knowledge gap by elucidating the specific Gnrh and Cck regulatory pathways that differentially control LH and FSH secretion from the pituitary gland of tilapia, providing novel insights into FSH’s unique regulatory mechanisms. Our findings demonstrate a precise differential localization of Gnrh receptors in tilapia, with Gnrhr1 specifically expressed in FSH cells and Gnrhr3 in LH cells. This specificity is critical for understanding the distinct regulation of gonadotropins. We propose that Gnrhr3 primarily mediates the increase in LH production and release, while Gnrhr1 is closely associated with FSH synthesis and secretion. This interpretation is strongly supported by our observations in female tilapia, where an increase in Gnrhr1 transcript levels correlated with elevated FSH release and the presence of vitellogenic follicles, whereas *lhβ* transcript levels coincided with mature follicles.

Genome duplication events have significantly influenced the duplication and nomenclature of Gnrh receptors in fish. Tilapia Gnrhr3 is similar to lumpfish, chub mackerel, medaka, and European seabass Gnrhr2ba1 (Madigou *et al.* 2000, Lumayno *et al.* 2017, Hodne *et al.* 2019, Andersson *et al.* 2023). In female tilapia, an increase in *gnrh* *r1* transcript levels correlated with an increase in FSH release and vitellogenic follicles, whereas *lhβ* transcript levels coincided with mature follicles. Our results corroborate previous results in other fish species: in medaka, *gnrh* *r2ba1* (similar to the tilapia Gnrhr3) was almost exclusively expressed in cells expressing *lhβ* (Hodne *et al.* 2019). In European sea bass, *gnrh* *r2ba1* transcripts are localized in all LH cells and only in a limited number of FSH cells (Madigou *et al.* 2000). In chub mackerel, a correlation between Gnrhr2ba1 (similar to GnRH-r3) and increased *lhβ* expression in the pituitary was observed during sexual maturation in both sexes (Lumayno *et al.* 2017). Of the four paralogous genes encoding Gnrh receptors in zebrafish, only Gnrhr2 (similar to tilapia Gnrhr3) was identified in the pituitary dataset and was uniquely expressed in the LH gonadotropes (Tanaka *et al.* 2024). Our results show that the specific receptor expression during development corresponds to the plasma level of a specific gonadotropin. LH levels fluctuate across various developmental stages, whereas FSH secretion is particularly elevated during the early vitellogenic stage, corresponding to the expression of *gnrh* *r1* and *gnrh* *r3* in the pituitary, supporting the accepted model in fish that FSH is responsible for follicle development and LH for final oocyte maturation and ovulation (Levavi-Sivan *et al.* 2010, Molés *et al.* 2020). This was also observed in rainbow trout and salmon, where basal secretion of FSH was high in previtellogenic and preovulatory fish but low in mature fish (Dickey & Swanson 2000, Vacher *et al.* 2000).

Our *in vivo* experiments revealed that Gnrh treatment in mature female tilapia elicited a significant increase in LH levels but only a limited, albeit statistically significant, elevation in FSH levels at 2 and 4 h post-injection. This finding in tilapia reinforces the established understanding that Gnrh’s effect on LH release is robust in fish, while its impact on FSH is considerably less pronounced (Levavi-Sivan *et al.* 2010, Molés *et al.* 2020). This differential responsiveness is consistent with observations in medaka, where FSH release does not strictly depend on Gnrh signaling (Takahashi *et al.* 2016), and in salmon, where sGnrh alone triggered LH release in mature females but did not stimulate FSH release at any stage (Ando *et al.* 2004). However, in mammals, unlike in fish, the effect of GnRH on FSH release is well established, with GnRH injections shown to increase FSH levels and FSH knockout inhibiting both FSH release and synthesis. In contrast, poultry exhibits a pattern similar to fish, where FSH release is less dependent on GnRH stimulation than on LH (Cerreta & McEntire 2024). Notably, fish and poultry are the only vertebrates with distinct cell populations for FSH and LH secretion (Hollander-Cohen *et al.* 2021), which probably contribute to differences in GnRH sensitivity. Furthermore, while GnRH has broader implications in the regulation of the release and expression of growth hormones (Marchant *et al.* 1989, Melamed *et al.* 1995), somatolactin (Kakizawa *et al.* 1997), thyroid-stimulating hormone (Roy *et al.* 2000), and prolactin (Seale *et al.* 2013), our *in situ* HCR data showing the absence of GnRH receptors in non-gonadotropin cells suggest that any additional effects of GnRH are likely indirect and potentially mediated through paracrine signaling.

Our research, both *in vivo* and *in vitro*, clearly demonstrates that LH secretion in response to GnRH is characterized by a higher peak amplitude and more rapid onset than that of FSH. In contrast, FSH exhibited a lower maximal response that persisted for a longer duration. In tilapia, each gonadotropin exclusively activates specific gonadal receptors (FSHR and LHR) without cross-reactivity or promiscuity (Aizen *et al.* 2012). This selective receptor activation aligns with the distinct biological roles of the gonadotropins. FSH is primarily responsible for vitellogenesis, a protracted phase that spans several days in tilapia, extends for months in carp, and can last for years in sturgeons. In contrast, LH facilitates the final maturation of oocytes and triggers ovulation, which is completed within a few hours in most fish species. Moreover, each phase is regulated by different environmental cues; the former depends on nutritional supply, whereas the latter depends on courtship. This specialization ensures that each hormone effectively supports its unique role in the reproductive cycle of fish.

Although both receptors exhibit a V-shaped indentation of the extracellular loop into the binding cavity, similar to the human homolog (Yan *et al.* 2020), they show notable structural variations in their binding sites, which may affect ligand affinity and binding conformation. *In vitro* data suggest that these receptors have varying degrees of affinity for GnRH analogs, which, in turn, affect gonadotropin secretion from distinct cell populations in tilapia. We hypothesized that the higher activity observed in tGnrhr3 towards native GnRHs in the transactivation assay compared to tGnrhr1 could be attributed to tGnrhr3’s wider binding cavity. This wider binding cavity may facilitate manipulation of the V-shaped indentation in the extracellular loop, which has been reported to play a crucial role in this type of receptor activation.

Our luciferase reporter gene assays provided direct evidence for distinct intracellular signaling pathways of the tilapia Gnrh receptors: Gnrhr1 (expressed in FSH cells) exclusively transduced its activity through the PKC/Ca2^+^ pathway, whereas Gnrhr3 (expressed in LH cells) mediated its intracellular signaling through both the PKA/cAMP and PKC/Ca2^+^ pathways, with high maximal responses. This corroborated previous findings that in tilapia, the signal transduction pathways that transduce the release of LH include the activation of both Gαq and Gαs (Levavi-Sivan & Yaron 1989*a*, *b*, 1992, 1993). Pituitary cAMP rises at proestrus, coinciding with the Gnrh preovulatory surge (Kimura *et al.* 1980), and is suppressed by Gnrh antagonist, indicating Gnrh drives this increase (Garrel *et al.* 2010). Gnrh pulsatility is partly decoded via the cAMP/PKA pathway (Cohen-Tannoudji & Avet 2012). Pathway-specific knockdown shows Gαq loss reduces Gnrh-stimulated *fshβ* mRNA levels, while Gαs knockdown reduces Gnrh-stimulated *lhβ* levels (Stamatiades & Kaiser 2018). These results suggest that in mammals, as shown here for fish, LH release is mediated by both the cAMP and PKC pathways, whereas FSH release is driven solely by the PKC pathway. Specifically, our results for tilapia corroborate and provide mechanistic insight into observations in mammals, where LH release is mediated by both cAMP and PKC pathways, and FSH release is primarily driven by the PKC pathway. This differential signal transduction mechanism underpins the distinct regulatory control observed for LH and FSH in this study.

The low responsiveness of FSH to Gnrh prompted us to investigate the alternative neuropeptides involved in FSH release. Recent analyses of tilapia, medaka, and zebrafish pituitaries revealed significantly higher *cck-rba* mRNA levels in FSH cells than in LH cells. Furthermore, studies on zebrafish and medaka have identified Cck as a hypothalamic FSH-releasing hormone (FSH-RH) (Hollander-Cohen *et al.* 2021, Hollander-Cohen *et al.* 2024, Tanaka *et al.* 2024, Uehara *et al.* 2024). Here, we demonstrate that CCK-Rs are not only directly expressed in FSH cells and regulate FSH secretion, but also influence the satiety of fish. Our *in situ* HCR experiments definitively demonstrated the explicit co-localization of *cck-* *rba* on FSH cells and its absence of expression on LH cells in double-labeled tilapia pituitaries. This finding is consistent with prior RNA-seq data showing abundant *cck-* *rba* receptors in tilapia FSH cells (Hollander-Cohen *et al.* 2021, Hollander-Cohen *et al.* 2024). We identified Cck-rba as a distinct third type of CCKR in teleosts, differentiating it from the mammalian CCKR1 and CCKR2. Further supporting a direct role for Cck, our pituitary clearing and immunofluorescence analysis revealed Cck-positive neurons originating in the brain that terminate specifically intertwined with FSH-secreting cells in the adenohypophysis. These direct neuro-pituitary contacts, characteristic of teleost fish and distinct from the median eminence system in other vertebrates (Ball 1981), strengthen the hypothesis that Cck directly binds to its receptor on FSH cells, thereby stimulating folliculogenesis. Such intense Cck immunoreactivity has also been observed within fibers of the PPD of goldfish (Himick *et al.* 1993) and recently in zebrafish (Hollander-Cohen *et al.* 2024) and medaka (Uehara *et al.* 2024). These findings reinforce the hypothesis that Cck binds to its receptor in the pituitary gland and stimulates FSH cells, which control folliculogenesis.

Our *in vivo* experiments highlighted the critical role of Cck in the fish pituitary. A single injection of tilapia Cck (100–500 μg/kg BW) induced a significant, dose-dependent increase in plasma FSH as early as 2 h post-injection and sustained up to 24 h. Importantly, we observed a corresponding increase in *fshβ* mRNA expression levels, while LH secretion and *lhβ* expression showed no significant difference or even a transient reduction. This specific stimulation of FSH by Cck in tilapia reveals a clear divergence from some findings in other species. For instance, in goldfish, Cck strongly stimulates LH release in sexually regressed fish (Himick *et al.* 1993). This could be attributed to antibody cross-reactivity, with high FSH levels in sexually regressed fish. In humans, where FSH and LH are secreted from the same cell, a direct stimulatory effect of CCK peptides was shown on both LH and FSH secretion (Oikonomou *et al.* 2007). Conversely, in rats, where gonadotrophs lack CCKRs (Fletcher *et al.* 2019, Hollander-Cohen *et al.* 2021), CCK can inhibit LH secretion, likely via GnRH neurons, while high doses in bulls elicited LH secretion (Vijayan *et al.* 1979, Kanematsu *et al.* 1990). However, we found that tilapia treated with increasing Cck concentrations showed increased plasma levels of FSH and *fshβ* mRNA expression levels, although we did not observe a significant difference in LH release or *lhβ* expression levels. Our results corroborate those in Mozambique tilapia, which, when subjected to prolonged starvation, showed a significant decrease in Cck immunoreactivity in the hypothalamus, pituitary, and intestine (Gouda & Ganesh 2025).

Beyond its long-recognized importance in mammalian gastrointestinal function and satiety, our current research in tilapia provides compelling, direct evidence that Cck plays a distinct role in regulating FSH secretion, thereby interacting metabolically with reproductive functions. This relies upon our previous study, which identified distinct hypothalamic regulation of LH and FSH in fish, demonstrating through *in vivo* and *ex vivo* calcium imaging in zebrafish that Gnrh primarily stimulates LH cells, whereas Cck preferentially activates FSH cells. The present study further solidifies this understanding by directly showing Cck’s role in FSH regulation in tilapia. This is also consistent with functional studies in zebrafish and medaka, where knockout of cck-rba results in significantly reduced gonad size and impaired female spawning (Hollander-Cohen *et al.* 2024, Uehara *et al.* 2024). These collective data firmly establish that Cck is a direct and critical regulator of FSH secretion in teleost fish.

A critical finding in the current study is that Cck significantly reduced food intake by 10% in the first 3 h after fish feeding. This direct demonstration of Cck’s role in satiety in tilapia aligns with its well-established function in mammals, where Cck significantly reduces food intake by inducing satiety and decreasing meal size (reviewed by Moran (2000)). Our results corroborate previous findings in other fish species that used mammalian CCK. In goldfish, both peripheral and central injections of CCK-8 suppress food intake (Himick & Peter 1994), and rainbow trout treated with CCK antagonists eat significantly more than the control fish (Gélineau & Boujard 2001). This consistent role in appetite regulation, when considered alongside its specific action on FSH secretion, highlights Cck’s unique position as a central metabolic gatekeeper influencing reproductive processes.

**Figure 8:**
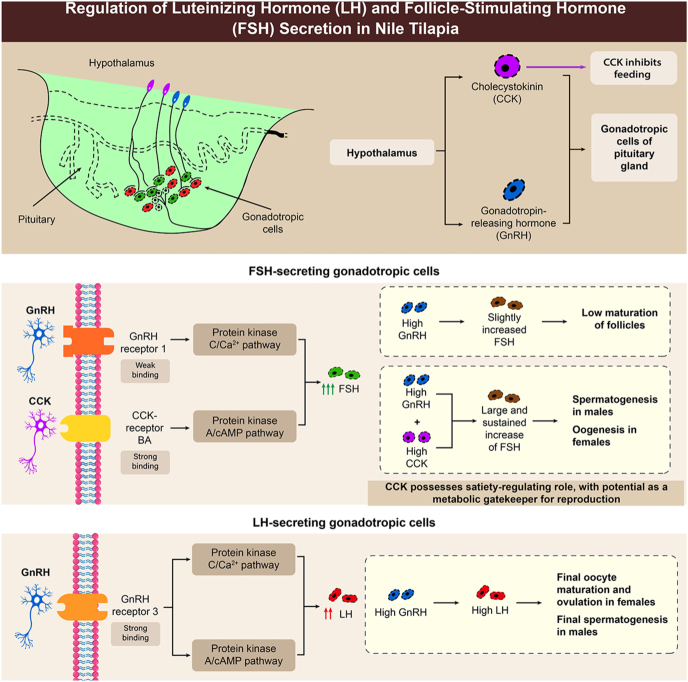
Summary model of Gnrh and Cck roles in tilapia reproduction. Gnrhr1 is expressed in FSH cells (juveniles), while Gnrhr3 localizes to LH cells across life stages. Gnrh primarily increases LH levels in mature females, with limited FSH response. Elevated FSH and Cck-rba expression during early vitellogenesis link Cck to energy availability. Cck strongly stimulates FSH release and concomitantly reduces food consumption, acting as a metabolic gatekeeper to align reproduction with nutritional status.

In summary, the comprehensive analysis in this study revealed two distinct hypothalamic pathways regulating LH and FSH, each linked to different stages of reproduction. Specifically, our data demonstrated that Gnrh primarily drives LH release, triggering final maturation and ovulation (as evidenced by the differential Gnrh-receptor localization and strong LH response). In contrast, FSH is regulated by both Gnrh and Cck, with Cck playing a more prominent role during gonadal growth (supported by its specific receptor expression in FSH cells, its strong stimulatory effect on FSH release, and its observed influence on food intake). This research establishes that Cck, a key regulator of satiety, creates a direct link between the metabolic status of fish and their reproductive function. This hypothalamic integration balances energy availability and reproductive capacity, consistent with a nutritional gating mechanism for gonadotropin release. Alternatively, Cck may independently regulate feeding and gonadotropin secretion. Such gating may delay or inhibit gonadal development under energy-deficient conditions, ensuring that reproduction occurs only when sufficient energy resources are available, thereby optimizing reproductive success concerning metabolic status (Fig. 8).

## Supplementary materials



## Declaration of interest

The authors declare that there is no conflict of interest that could be perceived as prejudicing the impartiality of the work reported.

## Funding

The project received funding from the US National Science Foundation, US–Israel Binational Science Foundation Joint Funding Research Grants (grant # NSF-BSF-1947541), and the Israel Science Foundationhttps://doi.org/10.13039/501100003977 (ISF) 1540/17.

## Author contribution statement

NM, MS, LHC, and BLS conceptualized the study. NM, MS, LHC, IA, and BLS contributed to methodology. NM, MS, and HM were responsible for investigation. IA was responsible for resources. NM, MS, IA, and BLS contributed to writing. BLS supervised the study.
