# Supplementary material for: Differential hypothalamic regulation of FSH and LH secretion from the fish pituitary by GnRH and CCK
Source: Reproduction. 2025 Oct 28;170(6):e250182. doi: 10.1530/REP-25-0182 (PMC12574605; doi:10.1530/REP-25-0182)
Supplement: Supplementary file 1 [file supplementary_materials.pdf]

REP-25-0182.R1-**Supplementary**

Differential Hypothalamic regulation of FSH and LH secretion from the Fish Pituitary by GnRH and Cholecystokinin

Naama Mizrahi*, Miriam Shulman*, Tomer Aiznkut, Ishwar Atre, Hadar Mor, Lian Hollander, Berta Levavi-Sivan

Table S1 Primer sequences used for RT-qPCR

Primer	5′ to 3′ sequence	Slope	Accession number	Range	R^2
18S-F	CGACCATAAACGATGCCAACTAG		XR_003218712.1	1052-1074	0.975
18S-R	GCACCACCACCCACAGAATC	−2.917		1308-1289	
tiEF1a-F	GCACGCTCTGCTGGCCTTT		NM_001279647	439-458	
tiEF1a-R	GCGCTCAATCTTCCATCCC	-3.49		688-667	0.99
lhβ-F	TGCTCCTTGCTCTGATGTTGA	−3.641	XM_025897714.1	72-92	0.999
lhβ-R	CCTTGGTGATGCAGTGTCCAC			254-234	
FSHβ-F	CTGTCGCCCAAAGAACATCA	−3.543	NM_001279743.1	94-113	0.998
FSHβ-R	AGGTCCCGCAGTCTGTGTTT			344-325	
tiGnRHr1-F	TATGGCCACGACCCTTCACAA	-3.027	NM_001279760.1	615-602	0.971
tiGnRHr1-R	TCGCCACACATGACCAATAGG			408-387	
tiGnRHr3-F	*TATTCACCATCCACTTCAGA*	−3.18	AY381299	1157- 1177	0.977
tiGnRHr3-R	TTAGAAAGCTGCTCCTCGGT			1344- 1364	
tiCCKr-F	TGTGAGAAATGAGACATCCATCA		XM_026145732.1	35-12	
tiCCKr-R	GATGTTTGTTGCCTCCAAGC	-3.3		132-111	0.98

Table S2 - List of exposed residues in the predicted binding pockets of tiGnRHR1 & tiGnRHR3.

Receptor	EXPOSED AMINO ACIDS IN BINDING POCKET
tiGnRHR1	P12 | P13 | L14 | D16 | W17 | E18 | A19 | S21 | F22 | T23 | A25 | A26 | R29 | M75 | V79 | D83 | M88 | K106 | L107 | M110 | S113 | A114 | H195 | F196 | L199 | Y200 | F258 | W262 | Y265 | Y266 | L268 | G269 | Y272 | W273 | L279 | R280 | T282 | P283| E284 | Y285 | H287.
tiGnRHR3	A50 | R51 | G52 | E53 | A54 | P55 | Q56 | L57 | R67 | V117 | D121 | A122 | N125 | I126 | M141 | K144 | L145 | M148 | Q197 | L200 | F201 | T216 | T217 | E227 | Y230 | N231| T234 | F235 | L238 | F239 | W302 | Y305 | Y306 | L308 | G309 | Y312 | W13 | D318| L319 | E320 | V323 | S324 | H325 | T328 | H329 |

	F332 | L336.

Table S3 – Comparison of EC50 values (nM) of GnRH-induced activation of tilapia GnRH receptors as presented in Figure 5. Serum responsive element (SRE)-Luc was used as a reporter gene following ERK/MAPK activation; cAMP responsive element (CRE)-Luc followed PKA activation; Nuclear Factor of Activated T-cells (NFAT) followed calcium signaling. Mean ± SEM.

**EC50 (nM)**	**Gnrh-r1**			**Gnrh-r3**		
	**SRE**	**CRE**	**NFAT**	**SRE**	**CRE**	**NFAT**
**GnRH1**	188.80±0.82	--	6.64±7.178	10.51±0.08	9.85±0.05	0.00005076±14.29
**GnRH2**	8.12±0.18	--	528.00±6.277	0.02±0.3	7.75±0.30	0.002247±11.65
**GnRH3**	93.29±0.05	--	0.12±9.916	2.71±0.18	10.51±0.14	0.2784±10.56
**sGnRHa**	19.89±0.08	--	3.16±8.501	0.39±0.05	2.38±0.30	1.63± 8.79

Table S4 - Number of samples in Figure 3 E-I

		Stage		
Fig. 3E	GSI	PE	8	
		EV	8	
		V	10	
		MF	12	
Fig. 3F	GTH		**FSH**	**LH**
		PE	8	7
		EV	7	9
		V	8	10
		MF	12	12
Fig. 3G	*gfh*		***fshb***	***lhb***
		PE	5	5
		EV	8	7
		V	8	10
		MF	10	7
Fig. 3H	*gth-r*		*fshr*	*lhr*
		PE	5	5
		EV	5	5
		V	7	7
		MF	9	6
Fig. 3I	*cck-rba*			
		PE	6	
		EV	6	
		V	8	
		MF	12	
